# A Unifying Model of Inflammatory Amplification: Insights From Colchicine Across Cardiometabolic and Infectious Disease

**DOI:** 10.7759/cureus.110588

**Published:** 2026-06-10

**Authors:** Amir B Rabbani

**Affiliations:** 1 Cardiology, University of California, Los Angeles (UCLA) Health, Los Angeles, USA

**Keywords:** atherosclerosis, colchicine, cytokine signaling, inflammation, innate immunity, nlrp3 inflammasome

## Abstract

Inflammation is a shared biological process underlying diseases that are traditionally viewed as distinct, including viral infections, coronary artery disease, and metabolic disorders. Despite differing clinical presentations, these conditions are driven by common immune-inflammatory pathways. This review describes how interactions between the innate and adaptive immune systems can become dysregulated, leading to a self-amplifying inflammatory cycle characterized by persistent immune activation, cytokine signaling, and tissue injury. Within this framework, inflammasome activation and immunometabolic dysfunction emerge as central drivers of disease. The role of colchicine is then examined as a therapeutic model that targets upstream components of the inflammatory amplification cascade. Finally, emerging therapeutic strategies are discussed, along with key gaps that may help guide more targeted approaches to treatment in the future.

## Introduction and background

Inflammation is increasingly recognized as a central mechanism underlying conditions traditionally viewed as distinct, including viral infections, coronary artery disease, and metabolic dysfunction [1]. Although these diseases differ clinically, accumulating evidence suggests that common immune-mediated processes contribute to tissue injury and disease progression across multiple organ systems [2]. Dysregulated inflammatory signaling has emerged as an important contributor to endothelial dysfunction, plaque instability, metabolic stress, and adverse cardiovascular outcomes.

At the center of this biology is a dynamic inflammatory response mediated by the body’s innate and adaptive immune systems. What begins as a protective host defense mechanism can evolve into a maladaptive process characterized by sustained immune activation, cytokine signaling, and tissue injury [3,4]. Diverse triggers, including viral infection, oxidized lipids, and metabolic stress, converge on inflammatory pathways involving inflammasome activation and immunometabolic dysfunction [5-8]. Persistent activation of these pathways may promote both acute inflammatory injury and chronic disease progression.

Colchicine, a well-established anti-inflammatory agent, provides a useful therapeutic model for examining these mechanisms [9]. By targeting upstream components of the inflammatory cascade, including neutrophil activation and NOD-, LRR-, and pyrin domain-containing protein 3 (NLRP3) inflammasome signaling, colchicine offers insight into how modulation of innate immune pathways may influence disease across multiple clinical settings [10,11].

This review outlines a conceptual framework in which dysregulated inflammatory signaling contributes to cardiometabolic disease and severe infection through self-amplifying immune activation. The interaction between innate and adaptive immunity is examined as a central contributor to disease pathogenesis, along with therapeutic strategies aimed at interrupting these inflammatory pathways. This narrative review was developed using literature searches performed in PubMed/MEDLINE, Embase, and Google Scholar, focusing on studies related to inflammation, NLRP3 inflammasome signaling, colchicine, cardiometabolic disease, and COVID-19 published between 2000 and 2026. Priority was given to mechanistic studies, translational investigations, randomized controlled trials, and contemporary review articles relevant to inflammatory amplification pathways. Therapeutic comparisons and pathway summaries are derived from findings reported in the cited literature, and no formal meta-analysis or independent statistical analysis was performed.

## Review

Innate and adaptive immunity in chronic inflammation

Overview of Inflammation

Inflammation is an evolutionarily conserved host defense response that protects against infection and promotes tissue repair [12]. Under normal physiologic conditions, inflammatory responses are self-limited. However, when inflammation does not resolve normally, it can lead to a dysregulated inflammatory state characterized by chronic low-grade immune activation [3]. This shift from acute defense to chronic dysfunction often stems from a breakdown in the regulatory crosstalk between the innate and adaptive immune systems. Effective immunity relies on the seamless coordination of these arms, beginning with the innate immune system’s ability to provide a rapid, non-specific response by recognizing conserved signals associated with infection or tissue injury [3-15]. These signals, derived from pathogens or damaged cells, are detected by pattern-recognition receptors (PRRs) such as toll-like and nucleotide-binding oligomerization domain (NOD)-like receptors [16]. Activation of these pathways leads to recruitment of effector cells such as macrophages, neutrophils, and dendritic cells, along with production of pro-inflammatory cytokines including interleukin (IL)-1, IL-6, and tumor necrosis factor (TNF) [12]. Beyond host defense, innate immune activation also shapes downstream inflammatory signaling and disease progression [13,17]. 

Dysregulation of Inflammation

The early innate response subsequently interacts with the adaptive immune system, forming a highly integrated and bidirectional network [18]. While innate immune activation initiates adaptive responses, adaptive immune cells modulate innate pathways through cytokine feedback. Effector cytokines such as interferon-γ and IL-17 promote macrophage activation and sustain inflammatory signaling. Regulatory mechanisms, including regulatory T cells, act to restrain excessive immune activation [19]. Disruption of this balance upregulates amplification of inflammatory signaling and impairs resolution of the inflammatory cascade. In chronic disease states, ongoing exposure to danger signals, such as oxidized lipids or tissue injury, drives persistent activation of innate immune pathways, including inflammasome signaling [1,6].

Innate Immunity and Initial Response 

As innate immune activation persists, it drives the adaptive immune system toward a sustained pro-inflammatory state, creating a self-reinforcing feedback loop [20]. When the crosstalk between innate and adaptive immunity becomes maladaptive, persistent antigen presentation and cytokine signaling involving IL-1β, IL-18, IL-6, and TNF-α amplify chronic inflammatory responses and impair normal resolution pathways [21]. This prolonged inflammatory activation contributes to endothelial dysfunction, plaque instability, myocardial injury, adverse ventricular remodeling, and progression of heart failure. In severe viral infections such as COVID-19, amplified cytokine signaling may further exacerbate thromboinflammatory injury and adverse cardiovascular outcomes, particularly in patients with underlying cardiometabolic disease.

**Figure 1:**
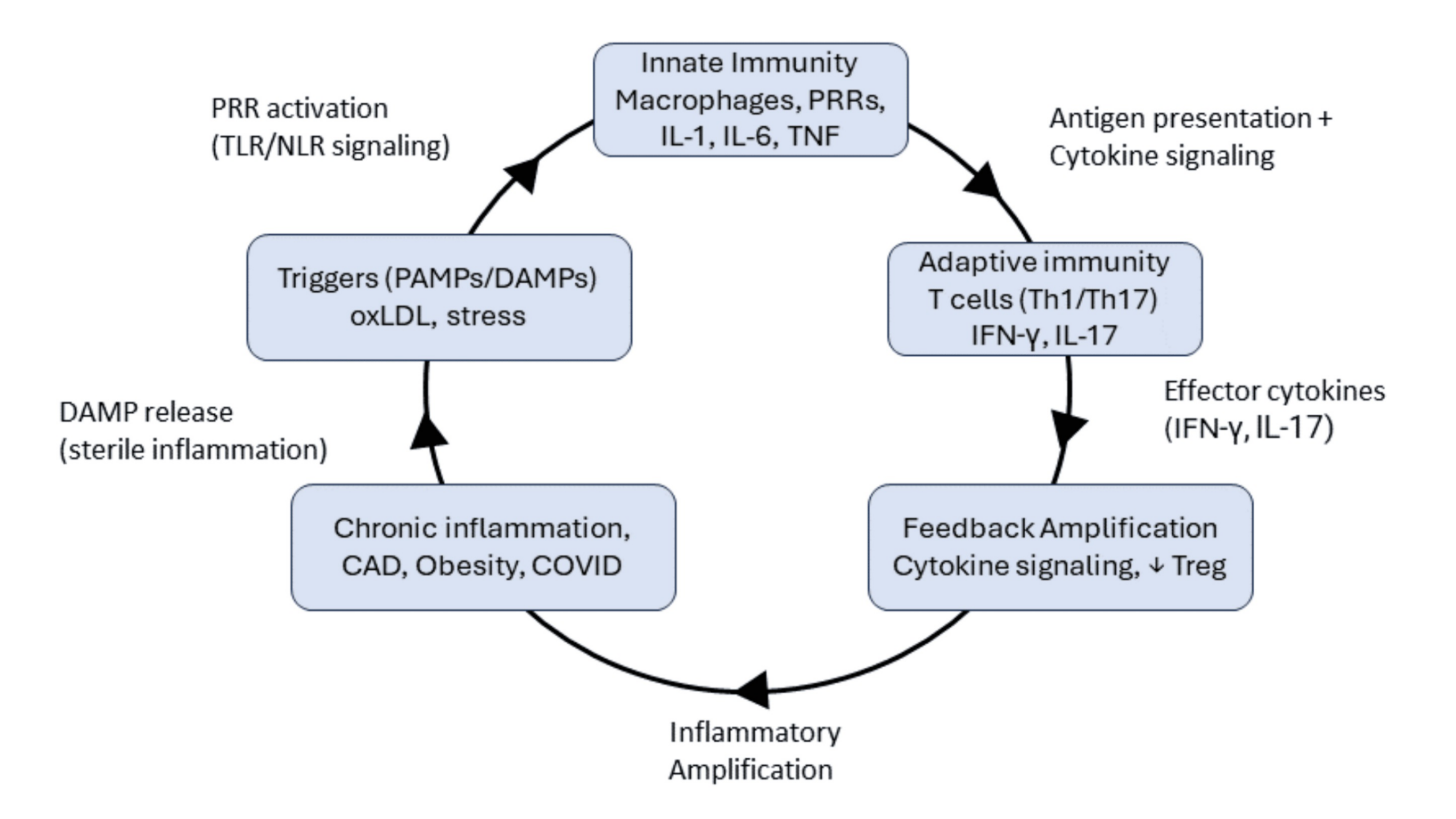
Self-amplifying inflammatory loop linking innate and adaptive immunity Triggers such as PAMPs and driven by DAMPs activate innate immune pathways through pattern-recognition receptors, leading to cytokine release and antigen presentation. This initiates adaptive immune responses, particularly Th1 and Th17 activation, which in turn reinforce innate signaling through effector cytokines. The resulting feedback amplification, along with reduced regulatory control, sustains chronic inflammation across disease states. PRR: pattern-recognition receptors; TLR: toll-like receptor; NLR: nod-like receptor; IL: interleukin, TNF: tumor necrosis factor; PAMPs: pathogen-associated molecular patterns; DAMPs: driven by damage-associated molecular patterns; oxLDL: oxidized low-density lipoprotein; IFN-γ: interferon gamma; CAD: coronary artery disease. Created by the author using BioRender (Science Suite Inc., Toronto, Canada)(https://BioRender.com/sqvztb3).

Another layer of dysregulation comes from trained immunity, in which innate immune cells undergo functional reprogramming following an initial inflammatory exposure and subsequently respond more aggressively to future stimuli [22]. Although distinct from antigen-specific adaptive immune memory, trained immunity may contribute to persistent inflammatory priming in chronic conditions such as atherosclerosis, obesity-related metabolic dysfunction, and heart failure. This persistent primed state may also help explain the exaggerated inflammatory responses observed in severe COVID-19 infection and long-COVID cardiovascular sequelae (Figure 1).

Together, these mechanisms provide a framework for understanding how atherosclerosis, metabolic disease, and severe viral infection may converge on common inflammatory signaling pathways [23]. 

Inflammasome-Mediated Cytokine Amplification

**Figure 2:**
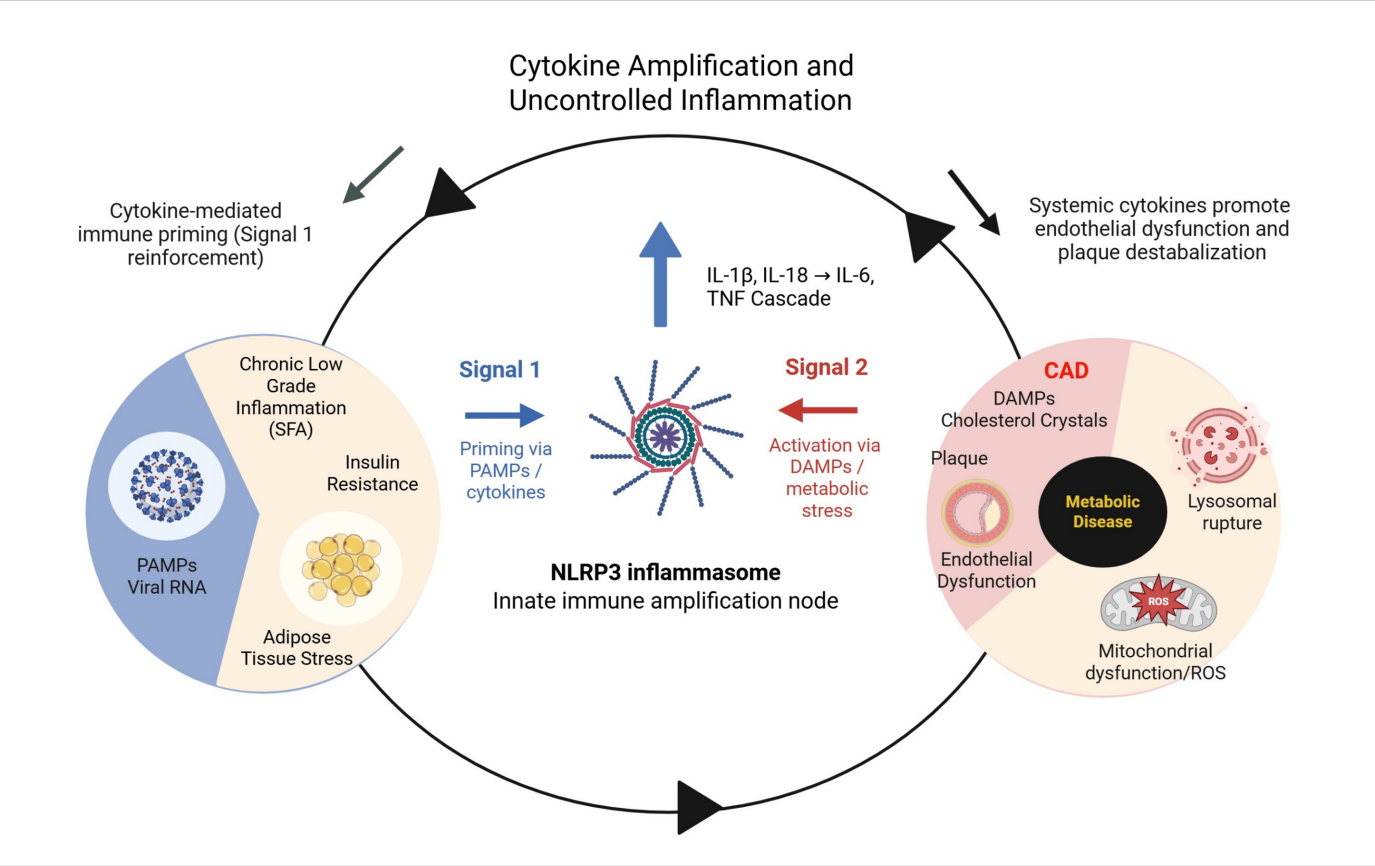
Schematic of inflammasome-driven cytokine amplification. Signal 1 (priming), triggered by pathogen-associated molecular patterns (PAMPs) or cytokines, prepares the innate immune system, while signal 2 (activation), driven by damage-associated molecular patterns (DAMPs) or metabolic stress, activates the NLRP3 inflammasome. This results in downstream cytokine release, including IL-1β and IL-18, with propagation through IL-6 and TNF signaling. These pathways reinforce immune activation and promote systemic inflammation, linking acute triggers such as viral infection with chronic conditions including atherosclerosis and metabolic disease. IL: interleukin, TNF: tumor necrosis factor; PAMPs: pathogen-associated molecular patterns; DAMPs: driven by damage-associated molecular patterns; NLRP3: NOD-, LRR-, and pyrin domain-containing protein 3; CAD: coronary artery disease; ROS: reactive oxygen species. Created by the author using BioRender (https://BioRender.com/sbr5lhc).

A central feature of this model is that inflammatory amplification does not occur as a single event, but through a linked two-step process involving priming (Signal 1) and activation (Signal 2) (Figure 2). The NLRP3 inflammasome sits at the center of this process, integrating upstream signals and amplifying them into downstream cytokine release [24,25]. Signal 1 is the priming step, typically triggered by pattern-recognition receptor signaling or cytokine activation through nuclear factor kappa B (NF-κB). This increases expression of inflammasome components, including NLRP3, along with cytokine precursors such as pro-IL-1β and pro-IL-18. Importantly, priming prepares the inflammasome for activation without triggering full cytokine release [26]. In acute settings, Signal 1 is driven by pathogens. In chronic disease, it is sustained by ongoing endogenous signals. Metabolic stress, oxidized lipids, and ongoing tissue injury generate low-level PRR activation and cytokine signaling, maintaining a primed state. In this setting, innate immune cells, particularly macrophages, remain primed and ready to respond, lowering the threshold for activation [27].

Impact of Chronic Inflammation on Disease Progression

Signal 2 is the activation step, triggered by cellular stress and injury, including extracellular ATP, cholesterol crystals, and reactive oxygen species, which drive assembly of the NLRP3 inflammasome [28]. These signals converge on common intracellular changes, such as ion flux, mitochondrial dysfunction, and lysosomal injury, that lead to inflammasome assembly and caspase-1 activation. Once activated, the inflammasome cleaves pro-IL-1β and pro-IL-18 into their active forms, initiating a downstream cytokine cascade [6]. These cytokines amplify inflammation both locally and systemically, driving further release of mediators such as IL-6 and TNF. At the same time, inflammasome activation can lead to pyroptotic cell death, releasing additional intracellular injury signals that further reinforce the response [29]. 

Furthermore, Signal 1 and Signal 2 do not act independently, but interact over time. In chronic cardiometabolic disease, persistent priming creates a baseline state of immune activation, so that even modest secondary stimuli can trigger exaggerated inflammatory responses [30]. This helps explain why patients with underlying conditions such as atherosclerosis or metabolic dysfunction are predisposed to amplified cytokine responses when exposed to additional stressors, including viral infection. When both signals are engaged in a primed environment, the response becomes amplified rather than simply activated. Cytokine production becomes self-reinforcing, with IL-1β and IL-18 sustaining upstream signaling and ongoing immune activation. This drives systemic inflammation, endothelial dysfunction, and tissue injury, and in severe cases can lead to uncontrolled cytokine release [31]. Thus, the NLRP3 inflammasome may serve as a mechanistic bridge between chronic low-grade inflammation and acute cytokine escalation across diverse disease states. 

Colchicine and disruption of the inflammatory amplification cascade

Given the central role of inflammasome-driven cytokine amplification across chronic inflammatory states, colchicine provides a useful therapeutic model for interrupting these upstream inflammatory pathways.

Role of Colchicine in Disrupting Inflammatory Amplification

**Figure 3:**
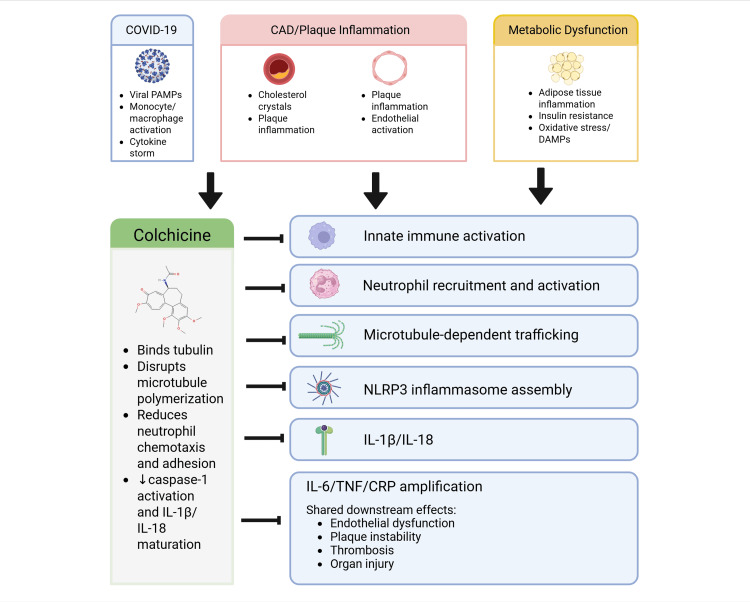
Mechanisms of colchicine action across shared inflammatory pathways. Diverse triggers, including viral infection, atherosclerotic plaque inflammation, and metabolic dysfunction, converge on innate immune activation. Colchicine acts upstream by disrupting microtubule-dependent processes, limiting neutrophil recruitment, inflammasome assembly, as well as IL-1β/IL-18 signaling. These effects attenuate downstream cytokine amplification, including IL-6 and TNF, and may reduce endothelial dysfunction and plaque instability. CAD: coronary artery disease; PAMPs: pathogen-associated molecular patterns; DAMPs: driven by damage-associated molecular patterns; NLRP3: NOD-, LRR-, and pyrin domain-containing protein 3; IL: interleukin; TNF: tumor necrosis factor; CRP: C-reactive protein. Created by the author using BioRender (https://BioRender.com/2p4e02y).

In this context, colchicine offers a promising therapeutic approach by targeting multiple steps within the inflammatory amplification cascade (Figure 3). Rather than acting on a single inflammatory pathway, colchicine disrupts crucial processes that drive this self-sustaining loop, providing a broader and more comprehensive strategy for modulating inflammation.

Mechanism of Colchicine Action

At its core, colchicine binds to tubulin and disrupts microtubule polymerization, a process that is central to immune cell function. Microtubules support intracellular trafficking and facilitate inflammasome assembly, making them vital to the activation of the innate immune response [9,32].

Early in the inflammatory response, colchicine limits neutrophil recruitment, adhesion, and migration, processes that contribute to inflammation in conditions such as atherosclerosis and acute infection. Neutrophils promote tissue injury and sustained inflammatory signaling through cytokine release and amplification of innate immune responses [33,34]. At low clinical doses, colchicine primarily dampens neutrophil chemotaxis and NLRP3 inflammasome activation while minimizing broader cellular toxicity. In contrast, higher colchicine concentrations may interfere with wider cellular structural functions, helping explain both its narrow therapeutic window and the ability of low-dose regimens to provide cardiovascular benefit with improved tolerability.

Inhibition of NLRP3 Inflammasome Activation

Furthermore, colchicine disrupts NLRP3 inflammasome assembly by impairing the microtubule-dependent transport of inflammasome components. This results in reduced activation of caspase-1 and decreased maturation of IL-1β and IL-18, cytokines central to the inflammatory amplification cascade [35].

As a result, colchicine reduces the production of pro-inflammatory cytokines such as IL-1β and IL-18, attenuating downstream cytokine signaling involving IL-6 and TNF-α. This suppression of cytokine amplification is reflected in reductions in systemic inflammatory markers, including C-reactive protein [23].

Modulating Inflammation Across Multiple Conditions

The therapeutic effects of colchicine are not confined to a single disease state. Diverse upstream triggers, including viral infection and atherosclerotic plaque inflammation, converge on shared inflammatory pathways involving innate immune activation and inflammasome signaling [36]. By targeting these pathways, colchicine may attenuate inflammatory signaling across a range of cardiometabolic and infectious conditions characterized by immune amplification.

Although colchicine is generally well tolerated at low cardiovascular doses, several important clinical limitations should be considered. Gastrointestinal intolerance, particularly diarrhea and nausea, is the most common adverse effect. Patients with significant renal or hepatic impairment may have reduced colchicine clearance and increased risk of toxicity, requiring dose adjustment and close monitoring. Drug interactions involving strong cytochrome P450 3A4 (CYP3A4) or p-glycoprotein inhibitors may also increase colchicine exposure. Rare but serious adverse effects include myopathy, cytopenias, and neuromuscular toxicity, particularly in elderly patients and those with underlying renal dysfunction.

In summary, colchicine acts at multiple points within the inflammatory cascade, from early innate immune activation to inflammasome signaling and downstream cytokine effects. These properties may help interrupt the feed-forward inflammatory loops that sustain chronic immune activation in a primed inflammatory state [22].

Current and Emerging Therapeutic Targets

Targeting Shared Amplification Points in Inflammation

The framework discussed earlier suggests that the most effective anti-inflammatory therapies may target key amplification points in the inflammatory cascade, rather than just addressing the initial trigger (Table 1). This is part of what makes colchicine an important proof of concept. In coronary disease, low-dose colchicine reduced cardiovascular events in both recent myocardial infarction and chronic coronary disease, showing that targeting upstream innate immune pathways can have clinical benefit across different settings [10,37]. Similar findings have been observed in inflammatory states associated with myocardial injury, including COVID-19, where colchicine has shown a signal for benefit in reducing cardiac injury and adverse outcomes [38-40]. 

**Table 1:** Therapeutic strategies targeting the NLRP3, IL-1β, and IL-6 inflammatory axis across multiple levels of the inflammatory cascade. Interventions range from upstream modulation of innate immune trafficking (e.g., colchicine) to direct inhibition of the NLRP3 inflammasome and downstream cytokine signaling, including IL-1 and IL-6 pathways. These therapies illustrate how targeting different points along a shared inflammatory pathway may influence disease processes across diverse disease states. Information summarized from previously published mechanistic studies and clinical trials involving NLRP3 inflammasome modulation and anti-inflammatory therapies, including COLCOT [10], LoDoCo2 [11], CANTOS [23], dapansutrile studies [41], and RECOVERY, and cardiovascular outcome trials involving statins, SGLT2 inhibitors, and GLP-1 receptor agonists. IL:  interleukin; CAD: coronary artery disease; RCT: randomized controlled trials; LoDoCo2: Low-Dose Colchicine 2 Trial; COLCOT: Colchicine Cardiovascular Outcomes Trial; NLRP3: NOD-, LRR-, and pyrin domain-containing protein 3; MCC950: selective NLRP3 inflammasome inhibitor; RECOVERY: Randomised Evaluation of COVID-19 Therapy; SGLT2: sodium-glucose cotransporter 2; GLP-1: glucagon-like peptide-1; CV: cardiovascular.

Target Level	Therapy	Mechanism	Key clinical evidence	Relevance
Microtubules/ innate immune trafficking	Colchicine	Binds tubulin; disrupts microtubule polymerization; inhibits NLRP3 assembly; reduces neutrophil chemotaxis/adhesion	LoDoCo2; COLCOT; mixed COVID-19 RCTs	CAD; COVID-19; metabolic inflammation
NLRP3 inflammasome	Dapansutrile (OLT1177)	Direct NLRP3 inhibition; reduces IL-1β/IL-18 release	Early-phase trials in gout/heart failure	Emerging therapeutic in cardiometabolic disease
NLRP3 inflammasome	MCC950 (experimental)	Potent selective NLRP3 inhibitor	Preclinical studies	Investigational
IL-1 signaling	Anakinra	IL-1 receptor antagonist	COVID-19 inflammatory phenotypes; rheumatologic diseases	COVID-19; systemic inflammation
IL-1β	Canakinumab	Monoclonal antibody targeting IL-1β	CANTOS trial (reduced cardiovascular events)	Atherosclerosis; residual inflammatory risk
IL-6 signaling	Tocilizumab; Sarilumab	IL-6 receptor blockade	RECOVERY and other COVID-19 trials	COVID-19; systemic inflammation
Downstream/pleiotropic	Statins; SGLT2 inhibitors; GLP-1 receptor agonists	Reduced vascular inflammation and cardiometabolic stress	Multiple CV outcome trials	CAD; metabolic disease

Moving Toward More Targeted Interventions

Building on this concept, a natural next step is to consider whether more targeted intervention at specific points in the cascade may offer greater benefit. One approach is to focus directly on the inflammasome. Dapansutrile, an oral selective NLRP3 inhibitor, has shown promising early results in gout and cardiovascular disease, targeting a central amplification point in the cascade [41,42]. More selective NLRP3 inhibition is also a concept worth exploring. Selective NLRP3 inflammasome inhibitor (MCC950), for example, has demonstrated that direct inhibition of NLRP3 can suppress IL-1β production with a high degree of selectivity in preclinical systems [43].

*Targeting Cytokine Signaling Downstream* 

Another potential strategy is targeting cytokine signaling further downstream of the inflammasome. The CANTOS trial, which involved IL-1β inhibition with canakinumab, showed that this approach reduced recurrent cardiovascular events, independent of lipid-lowering effects, although at the cost of increased infection risk [23]. Similarly, Anakinra has shown benefits in specific contexts, including COVID-19, supporting the blockade of the IL-1 pathway when immune activation drives disease severity [44]. Moving further downstream, IL-6 receptor blockade has proven effective as well. In the Randomised Evaluation of COVID-19 Therapy (RECOVERY) trial, tocilizumab improved outcomes for hospitalized COVID-19 patients, and both tocilizumab and sarilumab showed benefits in critically ill patients in the Randomized, Embedded, Multifactorial Adaptive Platform Trial for Community-Acquired Pneumonia (REMAP-CAP) trial [45,46]. These findings suggest that even after the inflammatory cascade is established, targeting dominant downstream cytokines can still offer clinical advantages.

Anti-Inflammatory Drugs Beyond the Traditional

Interestingly, therapies not typically viewed as primary anti-inflammatory treatments also fit within this framework. Statins, for instance, reduce vascular risk in part through their anti-inflammatory effects. The Justification for the Use of Statins in Prevention: an Intervention Trial Evaluating Rosuvastatin (JUPITER) trial showed that rosuvastatin lowered both low-density lipoprotein (LDL) cholesterol and high-sensitivity C-reactive protein [47]. SGLT2 inhibitors and GLP-1 receptor agonists also have broader cardiometabolic effects, including dampening inflammatory signaling. This helps explain why their benefits extend beyond just glucose lowering [48,49].

Therapies may therefore be better classified by their point of intervention within inflammatory signaling pathways rather than by disease category alone. Colchicine acts earlier in this process and has broader effects throughout the pathway. In contrast, therapies like NLRP3 inhibition or cytokine blockade tend to act at more specific points within that same cascade. The challenge moving forward is to identify the most effective point in the cascade to target for each clinical scenario, while ensuring that more selective approaches balance both effectiveness and safety.

Gaps in knowledge and future directions

While we have a conceptual framework for inflammation, several key uncertainties remain. It's still unclear what determines whether an initial inflammatory response resolves or becomes self-sustaining and amplified. The roles of priming and activation likely vary depending on the disease and can shift over time within the same patient. This variability makes it difficult to pinpoint the optimal time for intervention [6,24].

Challenges in Identifying Inflammatory Activity

Another significant challenge is the lack of clinically useful biomarkers to track inflammatory activity along the cascade. Common markers like C-reactive protein reflect downstream inflammation but offer limited insight into upstream drivers. More specific markers, such as IL-1, IL-6, or inflammasome activation indicators, are not yet practical for routine clinical use. Without a clear way to assess where a patient stands in the cascade, treatment remains largely empirical [50].

Clinical Trial Variability and the Need for Better Targeting

This uncertainty is also evident in clinical trial results. Colchicine has shown benefits in patients with recent myocardial infarction in the Colchicine Cardiovascular Outcomes Trial (COLCOT), but similar benefits were not observed in other settings, such as the Organization to Assess Strategies in Ischemic Syndromes (OASIS) [51,52]. These discrepancies likely go beyond trial design and point to a central limitation in our current approach: we still don’t have a reliable method to identify when inflammatory signaling is actively driving disease or which part of the cascade is most involved. As a result, therapies targeting a relevant pathway may not show consistent benefits, especially when applied at different stages of the disease process.

Bridging the Gap

Ultimately, there's a significant gap between our growing understanding of inflammatory pathways and how we apply this knowledge in clinical practice. Despite more clarity on the mechanisms involved, most therapies continue to be tested in broad populations, without considering the specific inflammatory pathways driving disease in each individual. A more effective strategy could be to align treatment with the dominant level of inflammatory signaling in each patient, rather than using a one-size-fits-all approach to treat diseases.

## Conclusions

Inflammation represents a shared biological process underlying conditions traditionally viewed as distinct, including viral infection, atherosclerosis, and metabolic disease. The interaction between innate and adaptive immune pathways contributes to a self-perpetuating inflammatory response that may drive both acute and chronic disease states. Central to this process is the NLRP3 inflammasome, which links chronic low-grade immune activation to amplified cytokine signaling and tissue injury.

Colchicine provides a useful clinical example of how targeting upstream inflammatory pathways may influence disease processes across multiple conditions. Its effects on innate immune activation and inflammasome signaling support the concept that common inflammatory mechanisms may underlie diverse cardiometabolic and infectious diseases.

Current limitations in biomarker identification and patient selection continue to restrict the ability to apply anti-inflammatory therapies in a more targeted manner. Further investigation into shared inflammatory pathways and mechanism-based therapeutic strategies may help improve the precision and effectiveness of future treatment approaches.
